# The landscape of alternative polyadenylation during EMT and its regulation by the RNA-binding protein Quaking

**DOI:** 10.1080/15476286.2023.2294222

**Published:** 2023-12-19

**Authors:** Daniel P. Neumann, Katherine A. Pillman, B. Kate Dredge, Andrew G. Bert, Caroline A. Phillips, Rachael Lumb, Yesha Ramani, Cameron P. Bracken, Brett G. Hollier, Luke A. Selth, Traude H. Beilharz, Gregory J. Goodall, Philip A. Gregory

**Affiliations:** a Centre for Cancer Biology, University of South Australia and SA Pathology, Adelaide, SA, Australia; b Faculty of Health and Medical Sciences, The University of Adelaide, Adelaide, SA, Australia; c Australian Prostate Cancer Research Centre - Queensland, Centre for Genomics and Personalised Health, Faculty of Health, School of Biomedical Sciences, Queensland University of Technology, Brisbane, QLD, Australia; d Flinders Health and Medical Research Institute, Flinders University, Bedford Park, SA, Australia; e Development and Stem Cells Program, Monash Biomedicine Discovery Institute and Department of Biochemistry and Molecular Biology, Monash University, Melbourne, Australia

**Keywords:** Quaking, epithelial-mesenchymal transition, alternative polyadenylation, Crosslinked immunopreciptation (CLIP) sequencing, 3’ untranslated region (3’UTR), RNA binding protein (RBP)

## Abstract

Epithelial-mesenchymal transition (EMT) plays important roles in tumour progression and is orchestrated by dynamic changes in gene expression. While it is well established that post-transcriptional regulation plays a significant role in EMT, the extent of alternative polyadenylation (APA) during EMT has not yet been explored. Using 3’ end anchored RNA sequencing, we mapped the alternative polyadenylation (APA) landscape following Transforming Growth Factor (TGF)-β-mediated induction of EMT in human mammary epithelial cells and found APA generally causes 3’UTR lengthening during this cell state transition. Investigation of potential mediators of APA indicated the RNA-binding protein Quaking (QKI), a splicing factor induced during EMT, regulates a subset of events including the length of its own transcript. Analysis of QKI crosslinked immunoprecipitation (CLIP)-sequencing data identified the binding of QKI within 3’ untranslated regions (UTRs) was enriched near cleavage and polyadenylation sites. Following QKI knockdown, APA of many transcripts is altered to produce predominantly shorter 3’UTRs associated with reduced gene expression. These findings reveal the changes in APA that occur during EMT and identify a potential role for QKI in this process.

## Introduction

Epithelial-mesenchymal transition (EMT) is a developmental process that facilitates cancer progression in epithelial-derived tumours, through the acquisition of mesenchymal features [1]. EMT can be initiated by extracellular cues that regulate extensive changes in gene transcription through induction of EMT transcription factors [2]. Of note, TGF-β signalling is a potent inducer of the transcription factors ZEB1 and ZEB2, which operate in a reciprocal feedback loop with miR-200 to drive dynamic changes in gene expression and cell state [3–6]. EMT also involves widespread changes in alternative splicing mediated by specific RNA-binding proteins (RBP) [7–10]. Recently, we and others identified a prominent role for the RBP Quaking (QKI) in promoting EMT-associated alternative splicing and the acquisition of mesenchymal cell traits through maintenance of mesenchymal splicing patterns [11,12]. QKI expression is elevated by loss of miR-200 during TGF-β driven EMT, where it binds to introns and results in the alternative splicing of hundreds of mRNA targets [12].

QKI is itself alternatively spliced to produce three major isoforms, QKI-5, QKI-6 and QKI-7, with distinct functions. By virtue of a nuclear localization signal, QKI-5 is localized in the nucleus, while QKI-6 and QKI-7 are predominantly expressed in the cytoplasm [13]. The nuclear QKI-5 isoform plays important roles in alternative splicing while the cytoplasmic QKI isoforms contribute to regulating many aspects of mRNA metabolism including mRNA stability, localization and translation [14–19]. Consistent with these functions, a subset of QKI-binding occurs within 3’UTRs during EMT [12]; however, it is currently unclear what the functional consequences of QKI 3’UTR binding are in mesenchymal cells.

The influence of alternative cleavage and polyadenylation (APA) on transcript diversity during EMT is largely unexplored. APA can produce transcripts with varying 3’UTRs or alterative last exons, which can impact mRNA stability, localisation, translation and in some cases regional translation of specific mRNAs [20]. RBPs can co-transcriptionally regulate APA leading to coordination of APA in a cell specific manner based on the relative expression or activities of these factors [21]. While 3’UTR shortening is prevalent in solid cancers and can promote expression of oncogenes [22,23], broader comparisons of APA and gene expression changes show no consistent relationship, indicating that these two mechanisms of gene regulation operate largely independently [24,25].

Here, we performed 3’ end anchored RNA sequencing (PolyA-Test RNA-seq or PAT-Seq) to quantitate the landscape of APA during EMT. Following TGF-β-induced EMT of human mammary epithelial cells (HMLE), we identified 79 events with significant APA, two-thirds of which resulted in longer 3’UTRs. By analysis of QKI CLIP-seq data, we identified a large proportion of QKI 3’UTR binding occurs adjacent to productive cleavage and polyadenylation (CPA) sites and found QKI knockdown in mesenchymal HMLE (mesHMLE) cells generally shortens 3’UTRs. These findings reveal the APA landscape during EMT and highlight a novel function for QKI as a regulator of APA.

## Results

### Alternative polyadenylation events regulated during EMT and by loss of QKI

While it is well established that post-transcriptional regulation plays a significant role in EMT, the extent of APA during EMT has not yet been explored. As the identification of shifts in CPA site usage requires a 3`end anchored RNA-Seq method, we performed PAT-Seq [26,27] on triplicate samples of human mammary epithelial (HMLE) cells and HMLE cells treated with TGF-β for 20 days to induce EMT (mesHMLE cells). We also performed PAT-seq on duplicate samples of mesHMLE cells transfected with siRNAs targeting QKI-5, a known regulator of alternative splicing in EMT, but with no recognized role in APA [12], generating on average ~ 8 million mapped reads per sample (Supplementary Table S1). Multidimensional scaling analysis showed distinct clustering of each sample type (Supplementary Figure S1A and Supplementary Table S2). This technique enabled us to identify changes in CPA site usage and poly(A) tail length during EMT as well as those regulated by QKI.

**Figure 1.**
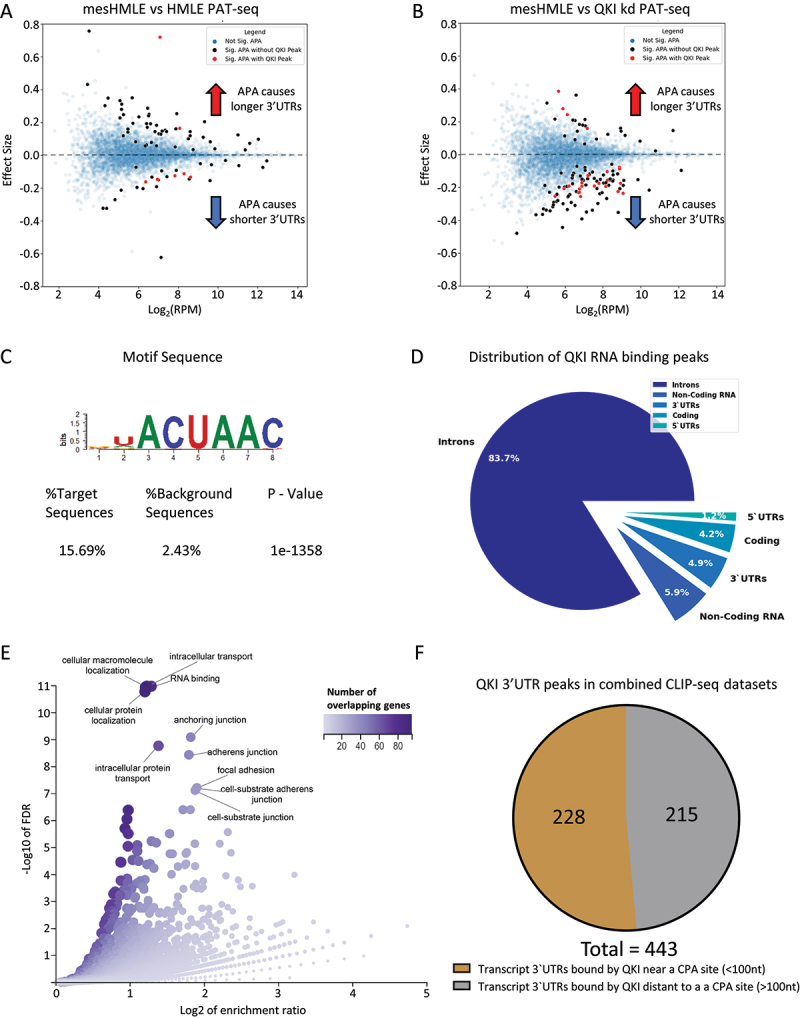
PAT-Seq uncovers widespread switching to distal cleavage sites after EMT and proximal sites after QKI knockdown. (A) MA plots for mesHMLE vs HMLE with APA effect size plotted against average expression across samples. (B) MA plot for mesHMLE vs mesHMLE + QKI knockdown. A positive effect size value indicates a shift to distal cleavage site usage, while a negative value indicates a shift to proximal sites. Blue dots indicate no significant change in APA, black dots indicate a significant change in APA and red dots indicate a significant change in APA and a QKI CLIP-Seq peak present in the 3’UTR of that transcript (in at least 3 QKI CLIP-Seq replicates). (C) The most enriched motif in the mesHMLE HITS-CLIP as determined by the software package HOMER. (D) Pie chart showing the distribution of QKI CLIP-Seq peaks by genomic location type. (E) Volcano plot of enriched GO terms from the set of 443 transcripts bound by QKI in at least 4 out of 7 separate CLIP-Seq experiments. Dot colour and size represents number of overlapping genes for that category. (F) Pie chart depicting the proportion of QKI 3’UTR targets with the QKI-CLIP peak centre within 100 nucleotides of an annotated cleavage site from the Poly A site atlas.

**Figure 2.**
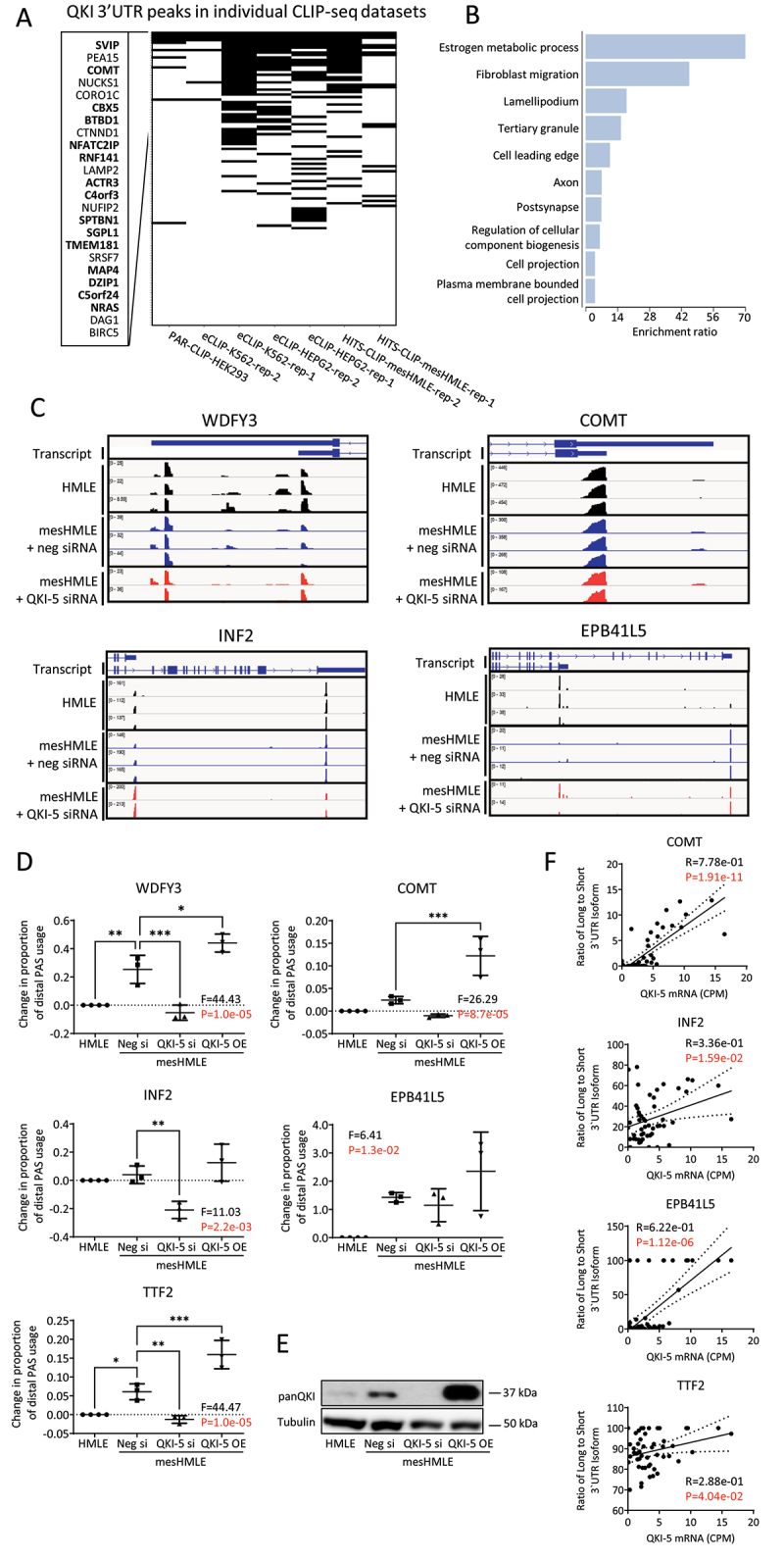
QKI 3’UTR binding often occurs near cleavage and polyadenylation sites. (A) Heat map comparison of QKI regulated APA events with QKI CLIP-seq datasets to identify APA events directly regulated by QKI. Genes with APA events and evidence of QKI binding in at least 3 QKI CLIP-seq datasets are indicated (*n* = 24). Where QKI binding sites are located within 100nt window either side of the APA site these are highlighted in bold. (B) Bar chart of enriched GO terms in the set of 24 genes. (C) Images of IGV tracks depicting change in cleavage site usage for selected genes. Individual tracks show depth-normalized coverage of each replicate PAT-Seq sample for HMLE, mesHMLE and mesHMLE + QKI knockdown. (D) Graphs showing the change in APA site usage for selected genes in HMLE (*n* = 4), mesHMLE (*n* = 3), mesHMLE + QKI-5 knockdown (*n* = 3) and mesHMLE + QKI-5 overexpression (*n* = 3), as determined by qRT-PCRs. For WDFY3 and COMT, proximal cleavage primers detect proximal and distal APA and are a measure of total mRNA. Error bars indicate standard deviation of the mean. Statistical significance determined by performing a one-way ANOVA (F-value and p-value indicated) followed by Sidak’s multiple comparison post-hoc test. *p < 0.05, **p < 0.01, ***p < 0.001. (E) Western blot of QKI-5 and Tubulin expression in representative samples from (D). (F) Scatter plot showing the ratio of the Long to short 3’UTR transcripts against total QKI mRNA in the Cancer Cell Line Encyclopedia breast cancer samples [35]. Line represents a linear regression model and the shaded area represents the confidence interval. Pearson correlation coefficients and associated p-values are shown with p-values <0.05 highlighted in red.

We verified the induction of EMT in the mesHMLE samples by confirming downregulation of epithelial markers E-cadherin and ESRP1 and upregulation of mesenchymal markers ZEB1, Vimentin, N-cadherin, and QKI (Supplementary Figure S1C, Supplementary Table S3 and 4). Transfection of mesHMLE with the QKI-5 siRNA led to reduced expression of each of the major QKI isoforms (Supplementary Figure S1B, consistent with its known role in self-splicing its own transcript [28,29]. Therefore, while QKI-5 is the predominant isoform in these cells, the QKI-5 siRNA treatment serves effectively as a total QKI knockdown as reflected in loss of total QKI protein (see Figure 2E). QKI knockdown did not change EMT markers, as previously observed (Supplementary Figure S1C) [12]. In each group of samples, the mean poly(A) tail length was similar indicating global changes in poly(A) tail length are not a feature of EMT or regulated by QKI (Supplementary Table S1). Likewise, tests for changes in poly (A) tail length of specific genes during EMT or after QKI knockdown did not uncover any significant events (Supplementary Table S5 and 6). In contrast, there were 79 genes with significantly affected APA during EMT, with 27 shortening events and 52 lengthening events (Figure 1A, Supplementary Table S7). QKI knockdown resulted in an opposing pattern of APA with 94 shortening and 25 lengthening events (total 119 genes, Figure 1B, Supplementary Table S8), suggesting QKI may promote a shift to distal CPA site usage during EMT. Examining the intersection in these datasets showed a small overlap between the statistically significant APA events (7 genes), although each of these events changed in opposing directions (Supplementary Figure S1D). These data suggest that QKI regulates a subset of APA events during EMT.

### QKI binds near productive cleavage and polyadenylation sites

Previously, we uncovered numerous QKI binding sites within introns by performing QKI-5 HITS-CLIP on mesHMLE cells [12]. These sites reflect QKI’s established role as a regulator of alternative splicing, where QKI-5 was found to directly bind and regulate alternative splicing of hundreds of mRNAs during EMT. However, a subset of peaks were found in 3’UTRs, suggesting that QKI may regulate some mRNAs through non-splicing mechanisms.

CLIP-Seq results can be greatly affected by the choice of methods and antibodies, and the choice of peak calling requires the selection of parameters that strike a balance between sensitivity and stringency [30]. Therefore, we sought to generate a list of high confidence QKI 3’UTR targets by pooling data from several QKI CLIP-Seq experiments. Along with our previously published QKI-5 HITS-CLIP in mesHMLE cells (rep-1) [12], we performed a second set of HITS-CLIP experiments using a combination of the same QKI-5 antibody and a panQKI antibody that recognizes all QKI isoforms (rep-2). This second QKI HITS-CLIP experiment is designed to capture all QKI isoforms and all potential 3’UTR targets of QKI. Consistent with our previous observations [12], we identified the known QKI motif to be the most highly enriched sequence within the peaks (Figure 1C) and found the majority of QKI binding occurred within introns (Figure 1D).

In this second QKI HITS-CLIP experiment, we identified 429 distinct mRNAs with QKI peaks within 3’UTR suggesting that QKI could regulate these transcripts (Supplementary Table S9). Compared with the QKI-5 HITS-CLIP rep-1, we observed an overlap of 157 peaks within 3’UTRs, which may be indicative of false positive or negative peaks or cytoplasmic-specific QKI interactions since the panQKI antibody recognizes both cytoplasmic and nuclear isoforms of QKI (Supplementary Figure S2A). To generate a list of higher confidence QKI 3’UTR targets, we expanded our analysis to include various published QKI CLIP-seq datasets. We analysed data from PAR-CLIP performed on HEK293 human embryonic kidney cells (PAR-CLIP-HEK293) and eCLIP performed on K562 chronic myelogenous leukemia cells (K562-rep-1 and K562-rep-2) and HepG2 hepatocellular carcinoma cells (HEPG2-rep-1 and HEPG2-rep-2) [31,32], constraining our analysis to peaks called within 3’UTRs (Supplementary Figure S2A). Using these datasets, we generated a unique list of regions that overlap with a peak in at least one QKI CLIP-Seq experiment (Supplementary Table S10). We subsequently tallied the number of experiments where a peak was successfully called for each unique region, and shortlisted regions containing a peak in at least four out of the seven experiments. This resulted in the identification of 622 peak-containing regions that were present within 443 distinct transcripts (Supplementary Table S10). To determine if these genes have functions that are relevant to epithelial plasticity, we performed gene ontology (GO) analysis and uncovered pathways related to EMT-associated properties, including cell junctions and focal adhesions (Figure 1E), supporting the notion that regulation of these mRNAs by QKI might be functionally important.

Next, we explored whether QKI binding within 3’UTRs may be involved in regulating APA. Recent analysis of the cytoplasmic isoform, QKI-6, found an enrichment of binding within 3’UTRs adjacent to the polyA signal (PAS) [17]. As the QKI isoforms share the same RNA-binding domain and recognize the same RNA-binding motif [33], we speculated that the nuclear isoform, QKI-5, might also bind near PAS and affect CPA. To assess this possibility, we compared the QKI CLIP-Seq data to the PolyA site atlas [34] to identify peaks that occur near productive CPA clusters. Of the 443 QKI 3’UTR targets, approximately half had annotated CPA sites within or nearby a QKI CLIP-Seq peak (Figure 1F). Although there was variability in the QKI CLIP-seq datasets, several of these showed QKI binding was primarily enriched in close proximity to the CPA site (Supplementary Figure S2B). These results suggest that a large proportion of QKI 3’UTR-binding occurs at productive CPA sites and uncovers a putative role for QKI in regulating alternative polyadenylation.

### A subset of alternative polyadenylation during EMT is controlled by QKI

To uncover APA events directly regulated by QKI, we compared the genes with altered APA after QKI knockdown to QKI 3’UTR CLIP-seq targets, identifying 24 genes that had altered APA and evidence of QKI binding in at least 3 separate experiments. This overlap was highly significant (*P* = 1.05 × 10^−9^, hypergeometric test), suggesting QKI directly regulates APA of this subset of mRNAs (Figures 2A and 1B). Gene ontology (GO) of these genes highlighted processes including lamellipodium, fibroblast migration and cell leading edge, indicating that QKI-regulated APA may contribute to changes in cell migration (Figure 2B), a feature prominently regulated by QKI in mesenchymal cells [12].

To validate the findings of the PAT-Seq, we performed qRT-PCR on select candidate genes with significantly altered APA during EMT or following QKI knockdown (Figure 2C). We also included the gene TTF2 that, although not significantly altered in the PAT-seq data, had strong evidence of QKI binding near to a productive CPA site. To detect a switch between mRNA isoforms with different cleavage sites within the same 3’UTR, we designed a primer set specific for the longer 3’UTR and another set that amplifies a common region. For cleavage sites in distinct 3’UTRs, we designed primers that were specific for each isoform. We found several genes with consistently altered APA after EMT, that changed in the opposite direction after QKI knockdown or were augmented after QKI-5 overexpression in mesHMLE cells, consistent with QKI controlling the choice between the alternative CPA sites (Figure 2D,E).

To determine whether the effects of QKI on APA occur more broadly, we examined expression of long and short 3’UTR isoforms of our candidate genes in breast cancer cell lines within the Cancer Cell Line Encyclopaedia [35]. Of the six transcripts where long and short 3’UTR are annotated, we found significant positive correlations between the ratio of long to short 3’UTR transcript and *QKI-5* mRNA for five of these suggesting that the balance between the long and 3’UTRs of these genes is dependent on QKI expression (Figure 2F).

### QKI regulates alternative polyadenylation of its own transcript

**Figure 3.**
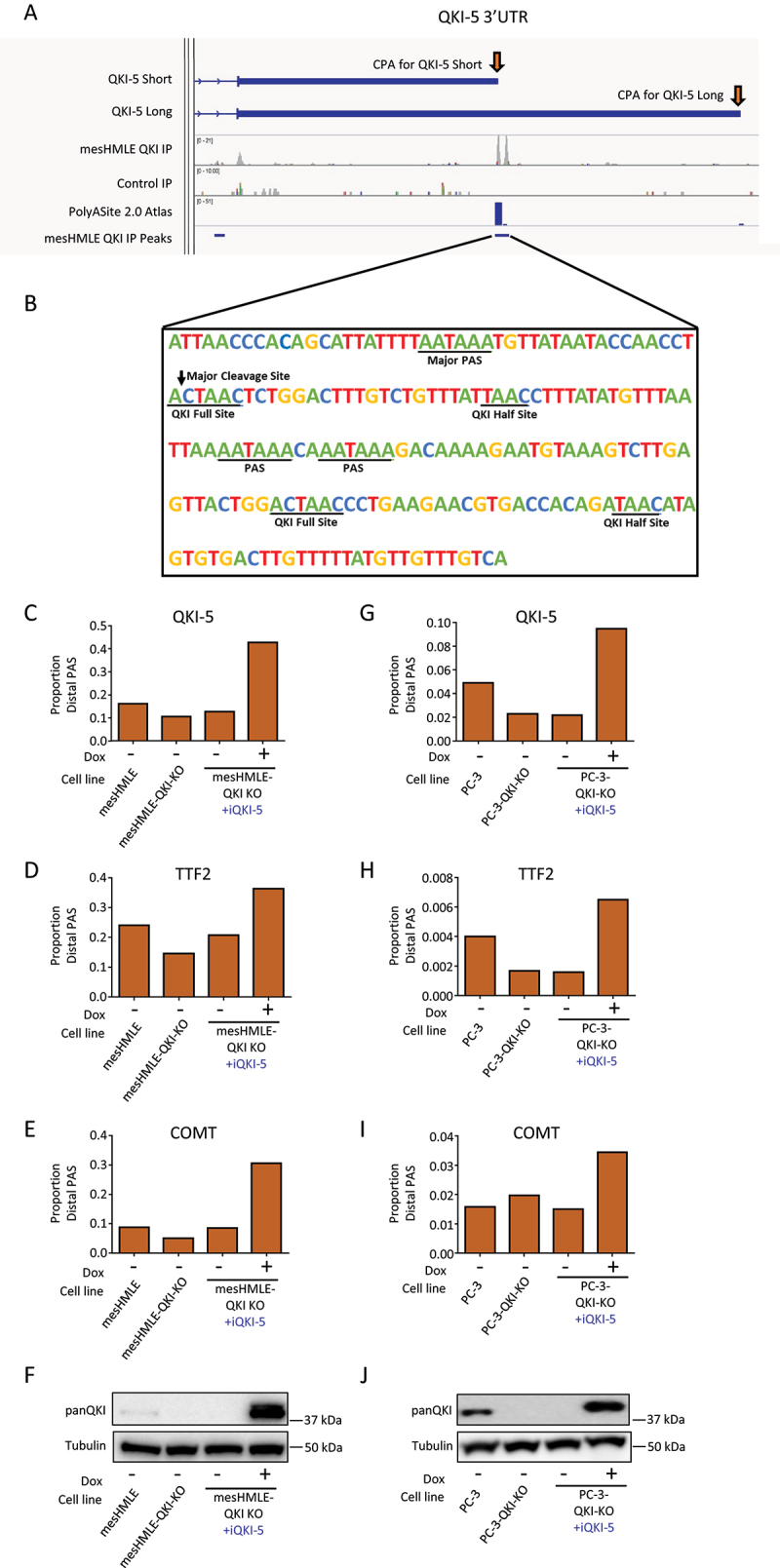
QKI-5 regulates its own APA to produce a transcript with a long 3’UTR. (A) IGV tracks depicting the location of QKI CLIP-Seq peaks in the QKI-5 3’UTR. (B) The nucleotide sequence around the predominant site of QKI binding in the QKI-5 3’UTR showing potential QKI binding and PAS sites. (C-E) Bar charts depicting the ratio of the isoform that uses the distal cleavage site to the isoform that uses the proximal cleavage sites for indicated genes determined by qRT-PCRs performed on mesHMLE, mesHMLE QKI knockout and mesHMLE QKI Knockout + inducible QKI-5, with and without 1 µg/mL doxycycline (Dox) treatment (*n* = 1), (G-I) and PC3-eLuc-GFP, PC3-eLuc-GFP QKI knockout and PC3-eLuc-GFP QKI knockout + inducible QKI-5 (*n* = 1). (F & J) Western blots of QKI and Tubulin protein performed on matched protein samples for (C-E & G-I).

Among the QKI-regulated APA genes, we identified two CLIP-seq peaks within the *QKI* transcript itself that overlapped canonical QKI-binding motifs and were located immediately downstream from an annotated CPA site for QKI-5 (Figure 3A). This region encompasses three distinct polyadenylation signals, one of which appears to be the predominant signal for polyadenylation of QKI-5 (Figure 3B). When examining the *QKI-5* 3’UTR in the UCSC genome browser, we found that many expressed sequence tags (ESTs) ended at the short 3’UTR terminus (QKI-5-Short) identified previously [12] or ~ 2kb upstream, with a smaller number ending at the annotated long 3’UTR terminus (QKI-5-Long) (Supplementary Figure S3A). At the QKI-5-Short terminus, the majority of ESTs ended at the QKI binding site downstream of the predominant PAS (Supplementary Figure S3B), which lead us to hypothesize that QKI may repress cleavage and polyadenylation at this location and utilize a downstream PAS to generate a longer QKI-5 3’UTR.

To confirm the existence of a *QKI-5* transcript with a longer 3’UTR and determine if QKI-5 protein induces its production, we examined expression of the QKI-5-Short and QKI-5-Long in a mesHMLE clonal cell line with CRISPR-generated QKI knockout. These cells had reduced QKI protein expression but retained QKI-5 mRNA, allowing us to modulate QKI-5 protein levels by exogenous expression and measure effects on endogenous QKI-5 mRNA. We transduced this cell line with a doxycycline-inducible QKI-5 expression construct and measured QKI-5-long and total QKI in wild-type mesHMLE, QKI knockout, and in QKI knockout cells with and without QKI-5 overexpression. Reduced QKI-5 protein in the knockout cells led to decreased production of QKI-5-Long mRNA, but not total endogenous QKI-5 mRNA, and a consequent decrease in distal to proximal PAS usage (Supplementary Figure S4A and 4C, Figure 3C,F). In contrast, overexpression of QKI-5 increased QKI-5-long while reducing total endogenous QKI-5 mRNA, leading to an increase in the distal to proximal PAS usage (Supplementary Figure S4A and 4C, Figure 3C,F). In these samples, we also verified that the proportion of distal to proximal PAS usage for Catechol-O-methyltransferase (*COMT*) and Transcription Termination Factor 2 (*TTF2*) are also influenced by QKI levels as observed previously (Figure 3D,E). To determine whether QKI autoregulation of 3’UTR length occurs in other contexts, we generated a second series of cell lines with the same QKI manipulations in the prostate cancer cell line PC-3. Similar to mesHMLE cells, we found QKI knockout reduced the distal to proximal PAS usage of QKI-5 and TTF2, while QKI-5 overexpression increased the distal to proximal PAS usage of QKI-5, TTF2 and COMT (Figures 3G-J, Supplementary Figure S4B and 4D). Collectively, these findings indicate that QKI-5 regulates its own APA, and the APA of other genes, to generate isoforms with generally longer 3’UTRs during EMT.

### QKI regulated APA increases 3’UTR length and gene expression

**Figure 4.**
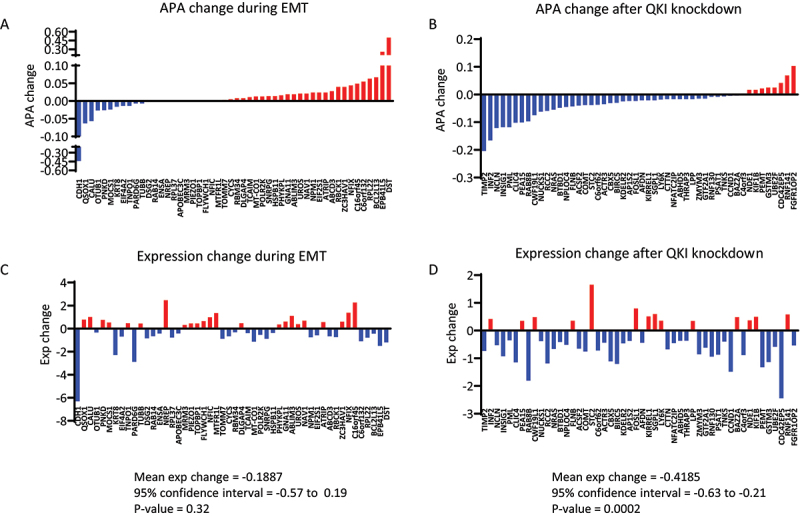
Correlation of APA changes with gene expression changed during EMT and following QKI knockdown. (A-B) Changes in APA during EMT of HMLE cells or following QKI knockdown in mesHMLE cells, aligned from most shortened to most lengthened 3’UTR. Only the top 50 differentially expressed genes with significant changes in APA (FDR <0.05) are represented (C-D) Corresponding changes in gene expression for APA affected genes. Blue bars indicate decreased APA and gene expression, and red bars indicate increased APA and gene expression. Statistical significance was measured using a one sample t-test on differentially expressed genes with significant APA change with mean expression change, confidence interval, and p-values shown.

To investigate the potential consequences of APA during EMT, we examined the relationship of APA to gene expression changes. While EMT was associated with large changes in gene expression (2203 genes, FDR < 0.05, Supplementary Figure S5A and Supplementary Table S3), only modest numbers of genes change expression following QKI knockdown (101 genes, FDR < 0.05, Supplementary Figure S5B and Supplementary Table S4), consistent with previous observations [12]. As the number of genes with significant expression changes in the QKI knockdown set was modest, we assessed the potential relationship between APA and gene expression using the top 50 differentially expressed genes that also had significant changes in APA. During EMT, while APA generally caused 3’UTR lengthening there was no consistent relationship between gene expression and APA (*p* = 0.32, Figure 4A,C). In contrast, QKI knockdown predominantly caused 3’UTR shortening and repression of gene expression (*p* < 0.002, Figure 4B,D). Collectively, these data suggest that QKI regulates a subset of genes during EMT associated with altered 3’UTR length.

## Discussion

To our knowledge, this study represents the first targeted examination of alternative polyadenylation (APA) during EMT using 3’ end anchored RNA sequencing. Here, we have shown that many transcripts display APA after EMT in human mammary epithelial cells, and present evidence that the mesenchymal splicing factor QKI regulates APA of several transcripts during EMT, including its own, to produce mRNAs with distinct 3’UTRs or last exons. This supports the existence of a mesenchymal-specific APA signature that QKI contributes to and warrants further investigation of how widespread this mode of regulation is in other contexts of EMT and cellular differentiation regulated by QKI [16].

With the PAT-seq method, we detected many APA events (~200) that were regulated during EMT and/or directly by QKI. While there was a small overlap between EMT and QKI knockdown regulated APA events, this is not entirely unexpected given that APA can be influenced by many RBPs, including those that are regulated during EMT [9,36]. However, validation of APA events by qPCR indicates that a larger fraction may be regulated in opposing directions in both scenarios. For instance, while the ratio of the long to short 3’UTRs of COMT and TTF2 increased during EMT and responded to QKI manipulation, these ratios were not significantly changed in the EMT PAT-seq dataset. This highlights limitations of the sensitivity of PAT-seq at the sequencing depth reported here (5–10 million reads per sample mapping to the genome).

While many APA events were detected to be regulated by QKI, we constrained our analysis to events where direct evidence for QKI 3’UTR binding was observed. Gene Ontology of this select gene subset revealed enrichment of EMT-associated properties indicating APA may cause functional changes consequential for EMT. For example, both Inverted Formin 2 (INF2) and Erythrocyte Membrane Protein Band 4.1 Like 5 (EBP41L5) have reported functions in controlling focal adhesion and actin dynamics that are important for cell migration [37,38]. Discovery of how these alternative 3’UTR sequences affect gene expression during EMT warrants further study.

During EMT, approximately two-thirds of APA events were associated with 3’UTR lengthening, while the opposite pattern occurred upon QKI knockdown in mesenchymal cells. Examination of the mRNA levels of APA-regulated transcripts during EMT revealed no correlation between gene expression and APA. In contrast, QKI knockdown generally decreased the expression of mRNAs whose APA it directly affected. Given QKI knockdown only influenced overall gene expression modestly, this suggests QKI-regulated APA may play a prominent role in generating more stable mRNA transcripts. Alternatively, as the binding of cytoplasmic forms of QKI is known to regulate the stability of specific transcripts [14,17,19,39,40], QKI may influence both APA and mRNA stability through different mechanisms. Although APA-induced 3’UTR shortening is a feature of cancer cells and can induce oncogene expression [22], broad associations between APA and gene expression are generally not observed [24,25,41]. APA and associated changes in 3’UTR usage can alter the localization and local translation of mRNA transcripts [42]. Considering the dynamic nature of epithelial plasticity, and the observation that altered protein localization can drive features of EMT in cancer [43], it may be that changes in APA of these mRNAs change the fate of their coded proteins, with potential consequences for protein folding, complex formation and localization. Indeed, protein complex formation and localization have been suggested to be 3’UTR-directed, with the long 3’UTR variant of Cluster of Differentiation 47 (CD47) specifically, interacting with SET Nuclear Proto-Oncogene (SET) protein and localizing it to the plasma membrane [44]. Further research is needed to determine what the functional consequences of QKI-directed APA are to the target mRNAs coded protein.

While the finding that QKI regulates its own APA has not been previously reported, the ability of QKI to self-regulate is known [28,29]. Indeed, the tendency of RBPs to auto-regulate is a well-established phenomenon, and auto-regulation of APA has been reported for other proteins of this class [45]. Whether QKI alternative polyadenylation serves as a mechanism of negative feedback or the switch to a longer 3’UTR affects the localization or function of QKI protein will require further investigation.

In summary, we have uncovered a novel role for QKI in regulating APA of a subset of transcripts during EMT. Along with its function in regulating EMT associated alternative splicing [12], these data demonstrate the diverse roles of QKI in regulating transcript maturation during EMT.

## Materials and Methods

### Cell culture

The immortalized human mammary epithelial cell line (HMLE) was cultured in HuMEC Ready Media (ThermoFisher) with HuMEC Supplement Kit (ThermoFisher) and passaged at a 1:5 ratio every 72 hours. The TGF-β-treated mammary epithelial cell line (mesHMLE) was derived by culturing immortalized human mammary epithelial cells (HMLE) in mesHMLE culturing media (DMEM/F12- Dulbecco’s Modified Eagle Medium: Nutrient Mixture F-12 supplemented with 5% Foetal Bovine Serum, 20 ng/ml EGF, 10 μg/ml insulin and 0.5 μg/ml hydrocortisone) with 2.5 ng/μL TGF-β1 for at least 14 days [46]. Once established, mesHMLE and mesHMLE QKI KO cells were all cultured in mesHMLE culturing media and passaged at a 1:10 ratio every 72 hours. PC-3 cells were grown in RPMI-1640 media supplemented with 10% Foetal Bovine Serum.

### Generation of QKI knockout and overexpression cell lines

QKI knockout cells were generated by CRISPR-Cas9 gene editing. For mesHMLE cells, a guide RNA sequence designed to target the first exon of the QKI gene (Supplementary Table 11) was combined with tracrRNA and Cas9 protein (Integrated DNA Technologies, AltR system) according to the manufacturer’s protocol and transfected using Lipofectamine RNAiMAX (ThermoFisher). Single clones were isolated following 72 hr of culture and screened for loss of QKI protein by Western Blot. For PC-3 cells, a QKI ko clone was derived after transfection of a LentiCRISPRv2 vector containing a BsmBI cloned gRNA targeting the first exon of QKI (Supplementary Table 11) and isolation of single clones following puromycin selection. QKI-5 overexpression cells were generated by transduction of mesHMLE and PC-3 wild-type or QKI ko cells, with either a pLX301-QKI-5 or pInducer20-QKI-5 lentivirus generated as previously described [12].

### Transfection of siRNAs

Transfections in this study were carried out with 20 nM of siRNA using Lipofectamine RNAiMAX and performed according to the manufacturer’s protocol (ThermoFisher). Transfection media was removed the following day after transfection and cellular material was harvested 72 hours after transfection. For PAT-seq samples, we used three untransfected replicates of HMLE cells, one untransfected (R1) and two negative control siRNAs (R2 and R3) replicates of mesHMLE cells, and two QKI-5 siRNA transfected replicates of mesHMLE cells. Sequences of siRNAs are provided in Supplementary Table 12.

### RNA isolation, cDNA synthesis and qRT-PCR

RNA was extracted using Trizol (ThermoFisher) following the standard manufacturer’s protocol. Complementary DNA was synthesized from 1 μg of RNA using the QuantitTect RT kit (Qiagen). Quantitative PCRs (qPCR) were performed in triplicate using the QuantiTect SYBR Green reagent (Qiagen) on a Rotor-Gene 6000 series thermocycler (Qiagen). Analysis was performed using the comparative quantitation feature in the Rotor-Gene software and qPCR assays were normalized to GAPDH expression to determine relative mRNA expression. To determine the proportion of distal polyadenylation site (PAS) usage for a given gene, the expression of the distal isoform is divided by the total expression of the gene. Sequences for the oligonucleotides used in the study are listed in Supplementary Table 11.

### Protein lysate purification and western blotting

Protein extracts were obtained by lysis of cells in 1×RIPA buffer (Abcam) containing cOmplete Mini, EDTA-free Protease inhibitor Cocktail tablets (Roche) and phosphatase (PhosSTOP EASYpack (Roche) inhibitors. Twenty micrograms of lysate were separated on an Invitrogen Bolt Bis-Tris Plus gel (Life Technologies), transferred to nitrocellulose, and probed with α-Tubulin (Abcam, ab7291), α-QKI-5 (Bethyl, A300-183A) or α-panQKI (Neuromab, N147/6) antibodies diluted 1:5000 in 5% skim milk. Proteins were detected by enhanced chemiluminescence (ECL) reagent (Pierce) using the ChemiDoc imaging system (Bio-Rad).

### QKI HITS-CLIP

The QKI-CLIP method was adapted from published methods [47], incorporating modifications from eCLIP [32] and iCLIP [48]. In brief, QKI was immunoprecipitated with a QKI-5 specific (Bethyl, A300-183A) or panQKI (Neuromab, N147/6) antibodies from crosslinked mesHMLE cells and sequencing libraries were prepared as detailed in the Supplementary Methods.

### PolyA-Test RNA-seq (PAT-seq)

PAT-seq libraries were prepared using 1 ug of total RNA as previously described [26,27]. Libraries were sequenced multiplexed on the Illumina NovaSeq 6000 instrument (Alfred Research Alliance Genomics Facility). Raw data was processed using the tail-tools pipeline [26]. The data from all samples was combined to identify 25,437 peaks associated with non-templated poly A-tracts. Of these 20,928 peaks and 73.3% of reads were in a 3’ UTR, 1349 peaks and 16.1% of reads were otherwise in an exon, 1202 peaks and 3.8% of reads were downstream of a non-coding RNA, 957 peaks and 0.7% of reads were in an intron, 403 peaks and 0.2% of reads otherwise antisense to a gene, 598 peaks and 5.8% of reads could not be related to annotated genes. Peaks were allowed up to 2000 bases downstream of the annotated 3’UTR end, but not inside another gene on the same strand. Statistically significant changes to gene expression, poly (A) tail-length and APA were calculated as previously described [49].

### Bioinformatic analyses

Bioinformatics for QKI HITS-CLIP was performed essentially as previously described [12] and detailed in Supplementary Methods. In brief, BAM files for samples that were prepared using the QKI-5 specific and panQKI antibodies were pooled together by strand. Peak calling was performed using the MACS2 with a cut off fold-enrichment value of 3 and a cut-off q value of 0.05 [50]. Motif analysis was performed using HOMER [51].

Publicly available QKI CLIP data [12,31,32] was used to generate the list of high confidence QKI-binding sites within 3’UTRs. The PolyA site atlas 2.0 [34] was used to identify productive cleavage and polyadenylation sites near QKI-binding sites. For coverage plots of QKI binding, the 3’UTR peaks for each QKI CLIP-seq dataset were plotted for the 1000nt spanning the highest expressed CPA in the PolyA site atlas (based on transcripts per million). The coverage plots of QKI binding were produced by selecting the 3’UTRs for each of the QKI 3’UTR peaks represented in each QKI CLIP-seq dataset. The highest expressed CPA in the PolyA site atlas (based on transcripts per million) for each 3’UTR was identified and QKI binding coverage was plotted for 500nt either side of the CPA.

Plots were generated using the Python packages Matplotlib and Seaborn, or GraphPad Prism. Motif logo figure was generated using the R package motifStack. Genome track figures were generated using Integrated Genomics Viewer [52] or the University of Santa Cruz Genome Browser [53].

Gene ontology analysis was performed using the tool WEB-based GEne SeT AnaLysis Toolkit (WebGestalt) using Over-Representation Analysis with the databases Biological Process, Cellular Component and Molecular Function [54].

Expression of the long and short 3’UTR transcripts of EPB41L5, PEA15, INF2, COL12A1, COMT and TTF2, and QKI-5 mRNA were obtained from the Cancer Cell Line Encyclopedia breast cancer samples [35]. The identities of the long and short 3’UTR transcript were determined by inspection of the Ensembl genome browser, with the transcripts used shown in Supplementary Table 13. Scatterplots were generated using the Python package, Seaborn, and Pearson correlation coefficients with associated *P* values were calculated using the Python package SciPy.

### Statistical analyses

A hypergeometric test was performed using the hypergeom.sf() function in the Python module scipy.stats to test for significant overlap between genes with QKI peaks and genes with APA in the PAT-Seq. This statistic takes into account the total number of expressed transcripts, which was determined to be 16,007 by counting the number of transcripts with a read count greater than 0 in the HMLE or mesHMLE RNA-Seq.

To test the changes in Distal PAS usage detected by qRT-PCR for statistical significance an ordinary 1-way ANOVA (*p* < 0.05) was performed on all samples for each APA event followed by a Sidak’s multiple comparison post-hoc test using GraphPad PRISM software.

## Supplementary Material

Supplemental Material

Supplemental_Fig_S1.pdf

Supplementary_Table_10.xls

Supplementary_Table_9.xls

Supplementary Table 3.xls

Supplementary_Table_4.xls

Supplementary_Table_5.xls

Neumann_SuppMethods.docx

Supplementary_Table_7.xls

Supplementary_Table_12.xls

Supplemental_Fig_S5.pdf

Supplemental_Fig_S2.pdf

Neumann_SuppTables.xlsx

Supplementary_Table_2.xls

Supplementary_Table_13.xls

Supplementary_Table_11.xls

Supplemental_Fig_S4.pdf

Neumann_SuppTables.xls

Supplementary_Table_8.xls

Supplementary_Table_1.xls

Supplementary_Table_6.xls

## Data deposition

The PAT-seq data and QKI HITS-CLIP data have been deposited into the Gene Expression Omnibus (GEO) with accession number GSE228932.
