# Supplementary figures and images for: The landscape of alternative polyadenylation during EMT and its regulation by the RNA-binding protein Quaking
Source: RNA Biol. 2023 Dec 19;21(1):199–209. doi: 10.1080/15476286.2023.2294222 (PMC10732628; doi:10.1080/15476286.2023.2294222)

Supplementary Figure 1

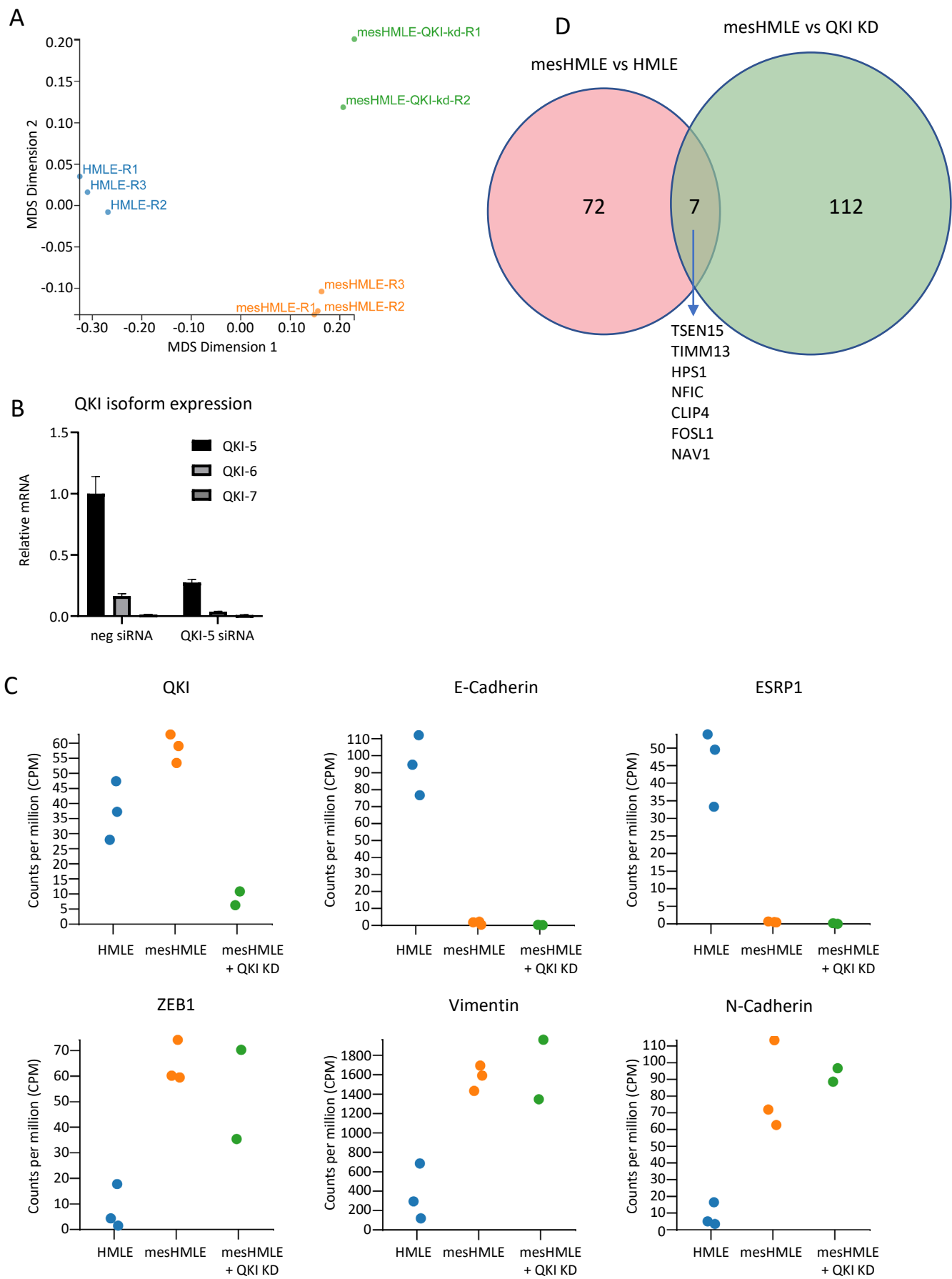

Supplement: Supplemental_Fig_S1.pdf [file KRNB_A_2294222_SM0807.pdf]

Supplementary Figure 5

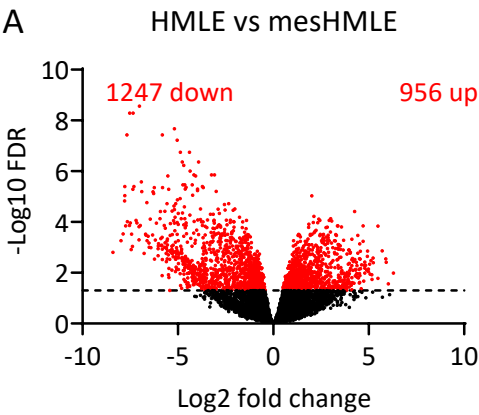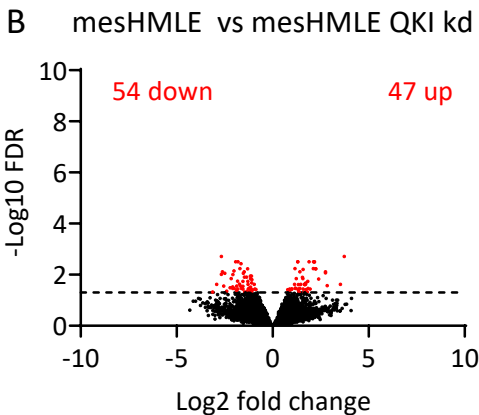

Supplement: Supplemental_Fig_S5.pdf [file KRNB_A_2294222_SM0798.pdf]

## Supplementary Figure 2

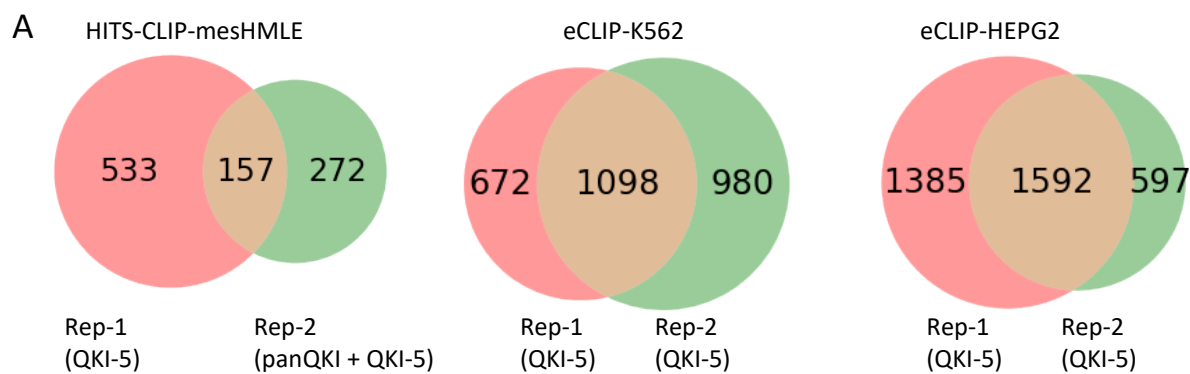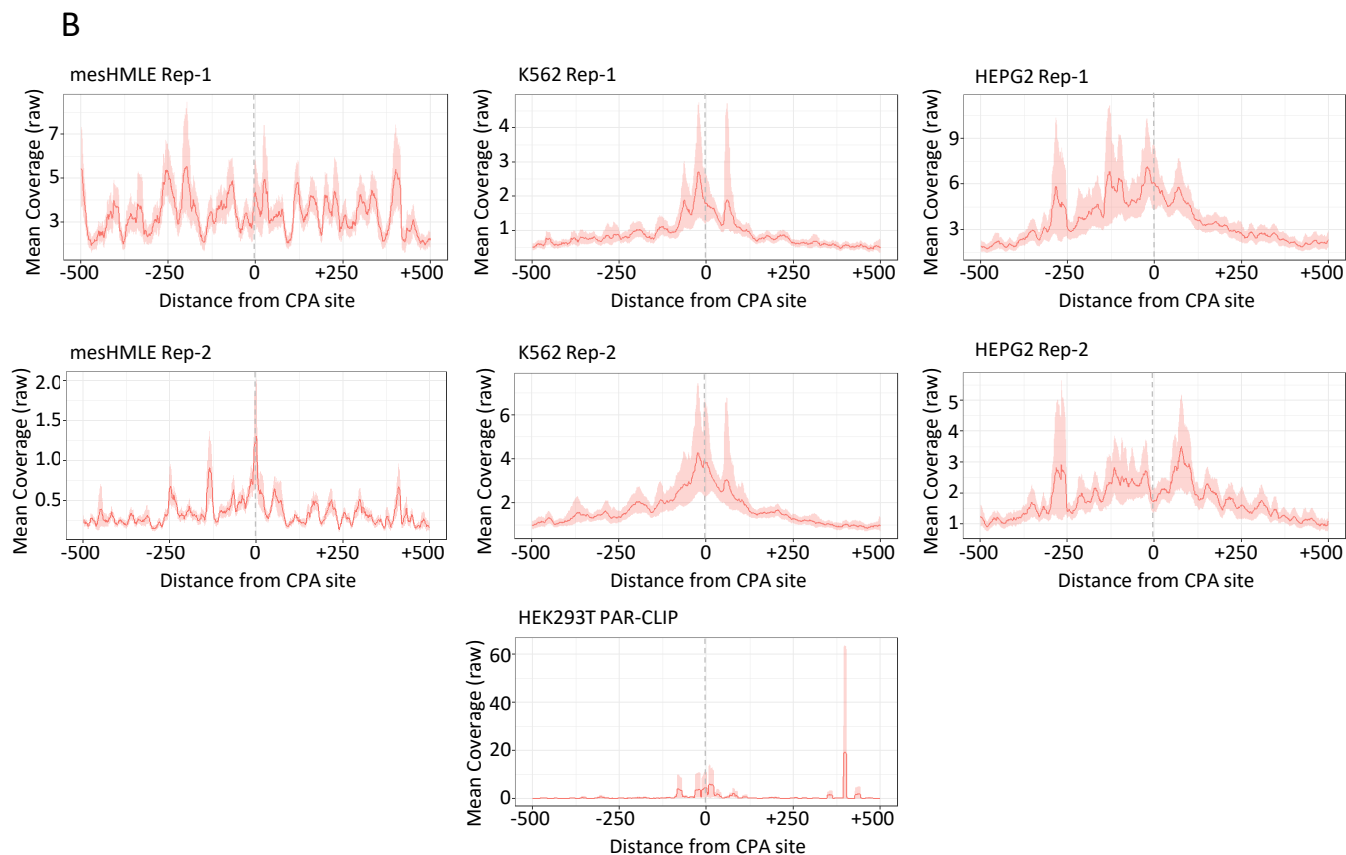

Supplement: Supplemental_Fig_S2.pdf [file KRNB_A_2294222_SM0797.pdf]

Supplementary Figure 4

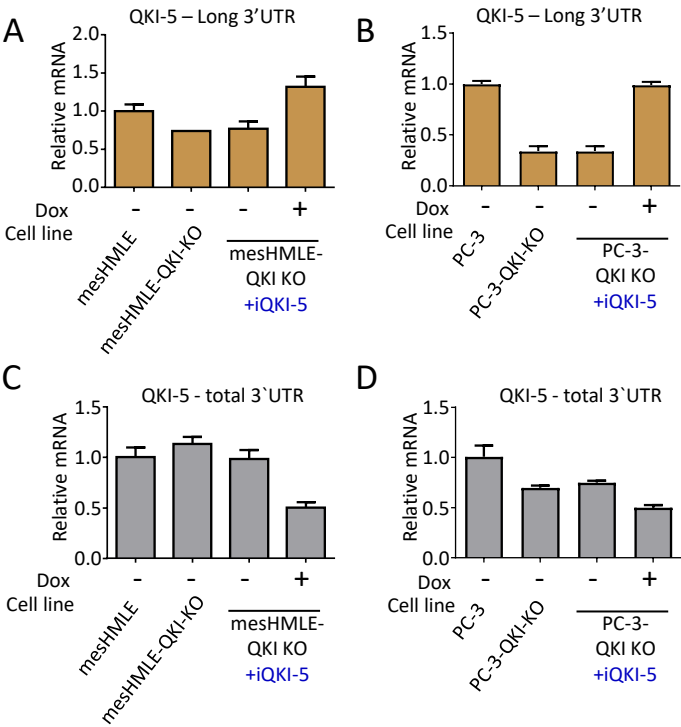

Supplement: Supplemental_Fig_S4.pdf [file KRNB_A_2294222_SM0792.pdf]
