# Supplementary material for: The landscape of alternative polyadenylation during EMT and its regulation by the RNA-binding protein Quaking
Source: RNA Biol. 2023 Dec 19;21(1):199–209. doi: 10.1080/15476286.2023.2294222 (PMC10732628; doi:10.1080/15476286.2023.2294222)
Supplement: Neumann_SuppMethods.docx [file KRNB_A_2294222_SM0801.docx]

**Supplementary Methods**

**QKI HITS-CLIP**

The QKI-CLIP method was adapted from published methods (Jensen and Darnell 2008), incorporating modifications from eCLIP (Van Nostrand et al. 2016) and iCLIP (Sutandy et al. 2016).

***UV crosslinking and preparation of lysates:***

MesHMLE cells were grown in 100mm plates to ~90% confluency, rinsed once with ice-cold PBS, and irradiated with 600 mJ/cm2 in ice-cold PBS using a UV Stratalinker-1800 (Agilent). Cells were collected by scraping, washed in PBS, and stored at -80°C as one pellet per plate. Each pellet was resuspended using 200 µl of 1 X QLB (1 X PBS, 0.3% SDS, 0.5% deoxycholate, 0.5% Igepal, EDTA-free Complete protease inhibitor cocktail (PIC; Roche, 11873580001) for 15 min on ice to liberate QKI from high molecular weight complexes, followed by addition of 400 µl of 1 X QDB (1 X PBS, 0.5% deoxycholate, 0.5% Igepal, PIC) and trituration by passing through a 21G needle and syringe 5 times. DNA was digested with 20 µl RQ1 DNAse (Promega, M6101) at 37°C for 10 min on a Thermomixer (750 rpm, Eppendorf). RNA was partially digested with RNase 1 (ThermoFisher, AM2295) by adding 6 µl of 1:50 diluted RNase 1 in 1 X PBS at 37°C for 5 min on a Thermomixer (750 rpm), then returned to ice. Lysates were centrifuged at 21,000 x g for 20 min at 4°C and supernatant transferred to a fresh tube.

***QKI Immunoprecipitation and complex purification:***

QKI-RNA complexes were immunoprecipitated from 500 µl of prepared lysate (1.1mg/ml) for 2 hr at 4°C using 5 µg anti panQKI specific antibody (NeuroMab 75-168, clone N147/6) bound to 100 µl protein G Dynabeads (ThermoFisher, 10004D), or QKI5 specific antibody (Bethyl, A300-183A) bound to 100 µl protein A Dynabeads (ThermoFisher, 10002D). A further 2% of IP input lysate was used as a size-matched input (SMin) adapted from Van Nostrand et al (Van Nostrand et al., 2016), with the addition of PAGE purification of the RNA prior to reverse transcription.  Bound QKI-5-RNA complexes were washed twice each consecutively with ice cold 1 X PXL (1 X PBS, 0.1% SDS, 0.5% sodium deoxycholate, 0.5% Igepal), 5 X PXL (5 X PBS, 0.1% SDS, 0.5% sodium deoxycholate, 0.5% Igepal), and 1 X PNK (50 mM Tris-Cl pH 7.5, 10 mM MgCl2, and 0.5% Igepal).

Replicate 1 samples were then labelled with P32 γ-ATP using T4 PNK (NEB, M0201L), the beads washed, and QKI-RNA complexes were eluted with 1 X Bolt LDS sample buffer (ThermoFisher) without reducing agent.

Replicate 2 samples were first treated with T4 PNK in the absence of ATP to dephosphorylate 3’ RNA ends followed by washes once each with 1 X PNK, 5 X PXL, then twice with 1 X PNK. A custom 3’ preadenylated linker (3’SRdeg_DNA /5rApp/GTACNNNNNNNNAGATCGGAAGAGCACACGTCT/3ddC/) was ligated to the RNA fragments on bead using RNA ligase I (NEB M0437M). Beads were washed as above then the RNA labelled with P32 γ-ATP using T4 PNK, washed again, then QKI-RNA complexes were eluted with 1 X Bolt LDS sample buffer (ThermoFisher) without reducing agent.

Both replicate QKI-RNA complexes and SMin controls were separated through Bolt 10% Bis-tris Plus gels (ThermoFisher) under non-reducing conditions then transferred to nitrocellulose (Schleicher&Schuell, BA-85). Filters were placed on a phosphor screen and exposed using a Typhoon imager (GE). 2 fragments of nitrocellulose were cut from each lane corresponding to the sizes of QKI monomer + covalently linked RNA, and QKI dimer + covalently linked RNA, and the RNA extracted and size-purified by PAGE as published previously (Pillman et al. 2018).

***Sequencing library construction:***

Replicate 1 libraries were constructed using NEBNext Multiplex small RNA library prep kit for Illumina with substitution of the 3' adapter with 3'SRdeg_DNA; /5rApp/GTACNNNNNNNNAGATCGGAAGAGCACACGTCT/3ddC/.

Replicate 2 libraries were constructed using a protocol modified from eCLIP (Van Nostrand et al. 2016) using custom adapters: 3' adapter 3'SRdeg_DNA, RT primer SR-RT; 5'-AGACGTGTGCTCTTCCGATCT-3', 5' adapter 5'SR_DNA; 5’P-GATCGTCGGACTGTAGAACTCTGAAC-3’SpC3/.

Products were amplified for 15 (Replicate 1), 16 (Replicate 2 CLIP) or 9 (Replicate 2 SMin) cycles using a common forward primer (NEBNext SR primer for Illumina) and barcoded reverse primers for each sample (NEBNext Index primers for Illumina). PCR products were purified using Qiagen Qiaquick PCR purification kit, separated on a 10% acrylamide (29:1) TBE non-denaturing gel, stained with SYBR Gold nucleic acid gel stain (ThermoFisher) and imaged on a ChemiDoc (BioRad). Products corresponding to an insert size of ~20 – 100 nt were excised from the gel and extracted by the “crush and soak” method as previously described (Jensen and Darnell 2008). Library quality and quantity was assessed by Bioanalyzer (Agilent), Qubit (ThermoFisher) and qPCR, pooled and sequenced on an Illumina NextSeq 500 (2 x 75bp).

***Bioinformatics and peak calling:***

Raw reads were trimmed and quality checked as described previously (Pillman et al. 2018). Using a custom python script, the spacer sequence (sequence: GTAC)  and degenerate barcode (8 bp) were clipped from the 3’ end of the read and the spacer sequence was added to the header of each read. Where the spacer sequence could not be identified from read 1, it and the barcode were identified from Read 2 instead using a second custom python script. Reads were mapped against the human genome (build: hg19), deduplicated and quality filtered as previously described (Pillman et al. 2018). Alignments were pooled to create single control and QKI files (Control: all control samples; QKI: all samples using either QKI-5 or panQKI-specific antibodies). Peak calling was performed as previously described (Pillman et al. 2018). Filtering was performed using a python script to remove low complexity peaks, discarding those that were supported by more than 10 reads but for which >25% of those reads mapped to an identical location.

**References**

Jensen KB, Darnell RB. 2008. CLIP: crosslinking and immunoprecipitation of in vivo RNA targets of RNA-binding proteins. *Methods Mol Biol* **488**: 85-98.

Pillman KA, Phillips CA, Roslan S, Toubia J, Dredge BK, Bert AG, Lumb R, Neumann DP, Li X, Conn SJ et al. 2018. miR-200/375 control epithelial plasticity-associated alternative splicing by repressing the RNA-binding protein Quaking. *EMBO J* **37**.

Sutandy FX, Hildebrandt A, Konig J. 2016. Profiling the Binding Sites of RNA-Binding Proteins with Nucleotide Resolution Using iCLIP. *Methods Mol Biol* **1358**: 175-195.

Van Nostrand EL, Pratt GA, Shishkin AA, Gelboin-Burkhart C, Fang MY, Sundararaman B, Blue SM, Nguyen TB, Surka C, Elkins K et al. 2016. Robust transcriptome-wide discovery of RNA-binding protein binding sites with enhanced CLIP (eCLIP). *Nat Methods* **13**: 508-514.
