# Supplementary material for: The landscape of alternative polyadenylation during EMT and its regulation by the RNA-binding protein Quaking
Source: RNA Biol. 2023 Dec 19;21(1):199–209. doi: 10.1080/15476286.2023.2294222 (PMC10732628; doi:10.1080/15476286.2023.2294222)

Supplementary Figure 3

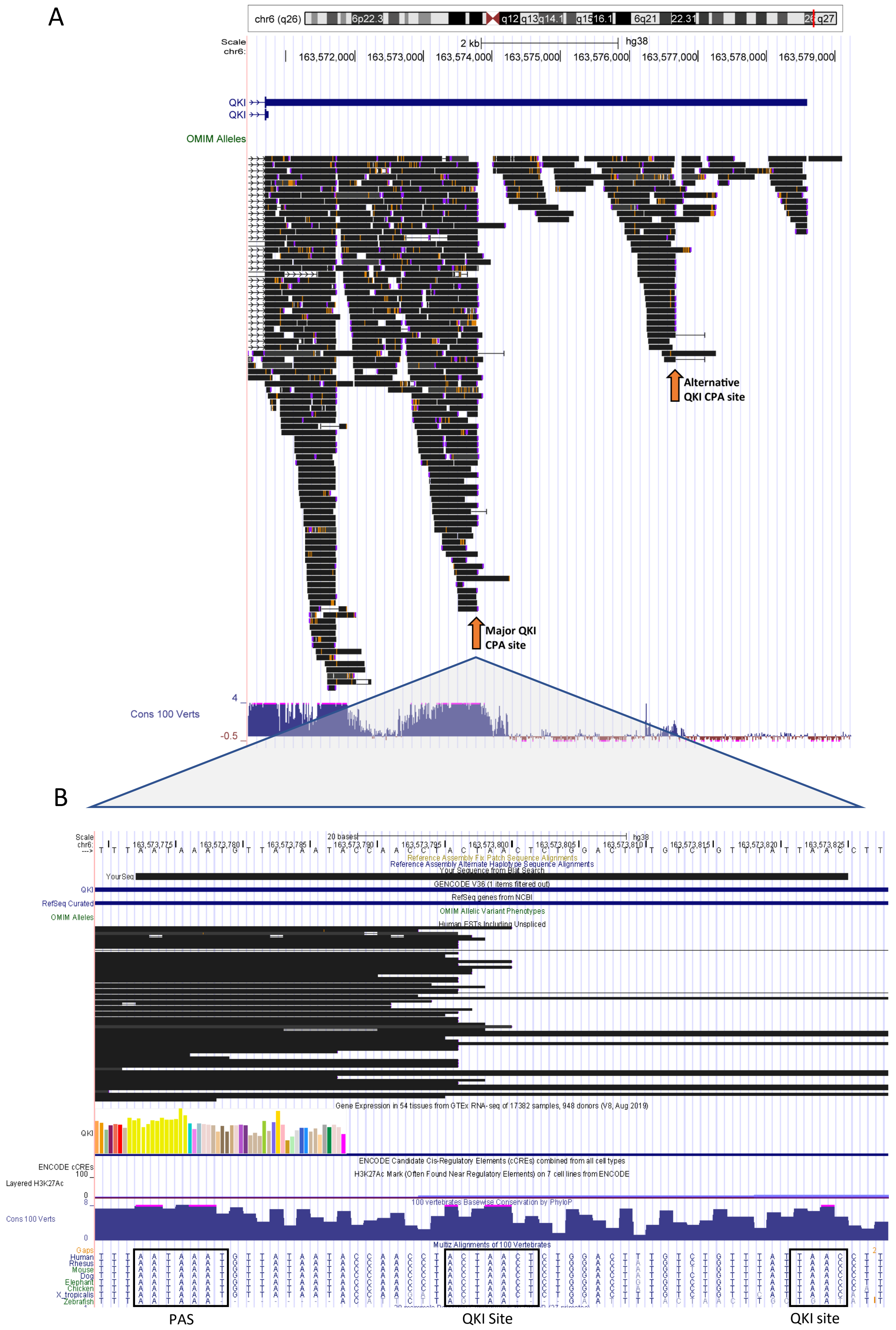

Supplement: Supplemental Material [file KRNB_A_2294222_SM4948.pdf]
